# Comparison of machine learning and validation methods for high-dimensional accelerometer data to detect foot lesions in dairy cattle

**DOI:** 10.1371/journal.pone.0325927

**Published:** 2025-06-27

**Authors:** Muhammad Usman Riaz, Luke O’Grady, Conor G. McAloon, Finnian Logan, Isobel Claire Gormley

**Affiliations:** 1 School of Mathematics and Statistics, University College Dublin, Belfield, Dublin, Ireland; 2 School of Veterinary Medicine, University College Dublin, Belfield, Dublin, Ireland; 3 Centre for Veterinary Epidemiology and Risk Analysis, School of Veterinary Medicine, University College Dublin, Dublin, Ireland,; 4 Royal (Dick) School of Veterinary Studies, Easter Bush Veterinary Centre, Roslin, Midlothian, Scotland; Universidade Federal de Mato Grosso do Sul, BRAZIL

## Abstract

Lameness is one of the major production diseases affecting dairy cattle. It is associated with negative welfare in affected cattle, economic losses at the farm level, and adverse effects on sustainability. Prompt identification of lameness is necessary to facilitate early treatment, enhance animal welfare, and mitigate short and long-term production impacts associated with the disease. In recent years, automated detection systems have emerged as a potential solution for identifying early signs of lameness. Among these systems, accelerometers have been widely adopted, as they continuously capture data on animal movement. Analyzing accelerometer data is challenging due to its wide, high-dimensional structure as it has many features and typically much fewer animals or samples, reducing the utility of many machine learning (ML) models and increasing the risk of overfitting. To handle this, researchers often summarize accelerometer data into indices like step counts, which simplifies analysis but may sacrifice important details needed for accurate prediction of lameness. Dimension reduction techniques, such as principal component analysis (PCA) and functional principal component analysis (fPCA), offer solutions by reducing the dimensionality of the data while retaining key information and allowing for the application of a broader set of ML approaches. Using data containing 20 thousand recordings from 383 dairy cows in 11 dairy herds, this study evaluated the effectiveness of ML methods in detecting foot lesions in dairy cows using accelerometer data, with a focus on dimensionality reduction approaches and cross-validation strategies. Our study offers practical insights for the dairy industry by highlighting the potential benefits of combining dimensionality reduction with cross-validation strategies to improve the performance of ML methods applied to wide accelerometer data. In addition, our study highlights the impact and importance of using data from independent farms. A by-farm approach to cross-validation will likely give a more robust, realistic estimate of general model performance.

## Introduction

Lameness is a significant production disease impacting dairy cattle. It is associated with negative welfare in affected cattle due to pain of prolonged duration [1]; economic losses at farm level due to production, treatment and labour costs [2–6] and adverse effects on sustainability, since affected animals are less productive and are often prematurely culled from the herd [7]. Lameness events may result in chronic changes to the foot structure, which predispose cows to future lameness events [8]. Therefore, prompt identification of lame animals is necessary to facilitate early treatment, enhance animal welfare, and mitigate both short- and long-term production impacts associated with the disease [9]. Classical lameness detection methods commonly used in the industry depend on visual assessment which uses assessment criteria to interpret gait and walking patterns of cows according to a series of score criteria or descriptions relating to gait abnormality severity. Visual mobility scoring (MS), one of the most frequently used methods, has some major limitations, including labor requirements and underestimation of lameness prevalence by farmers [10]. Recently, our research group demonstrated that MS had limited sensitivity and specificity for the detection of foot lesions in dairy cows [11].

In recent years, automated detection systems have emerged as a potential solution for identifying early signs of lameness [11,12]. Among these systems, accelerometers have been widely adopted, as they can continuously capture data on movement patterns, such as walking, standing, and lying behavior, without manual intervention [13]. Published studies report varying degrees of accuracy and reliability, with many models struggling to maintain consistency across different herds or environmental conditions [14–16]. In addition to advances in sensor technology, the development of advanced statistical tools, including ML-based automated detection models for lameness, is on the rise, as shown by recent surveys [17–20]. However, studies using modern ML approaches have yielded mixed results [21–24] with limited predictive success despite exploring features like gait, lying time, sleeping, and ruminating behaviors.

Most of the ML models used to date in animal health monitoring have been trained using the existing industry standard outcomes, such as MS, as the ground truth. However, this approach faces several limitations. As mentioned above, MS relies on subjective visual assessments by human evaluators, making it prone to inter-scorer variability (differences between evaluators) and intra-scorer variability (inconsistencies in the same evaluator’s scoring over time). These inconsistencies limit the accuracy and repeatability of MS as a reliable source of training outcomes. As a result, ML models trained using MS as the true classification inherited these errors, causing unreliable predictions when applied to real-world datasets. Although not free from limitations, a more robust alternative would be to train ML models on more objective health data, such as confirmed foot lesion detection collected through clinical examinations or veterinary diagnoses.

An additional challenge faced in analyzing accelerometer data arises from its wide structure, with many features for relatively few animals or samples, reducing the utility of many ML models and increasing the risk of overfitting. To handle this, researchers often summarize such accelerometer data into indices like step counts, which simplifies analysis but may sacrifice important details needed for accurate predictions. Dimension reduction techniques, such as principal component analysis (PCA) and functional principal component analysis (fPCA), offer potential solutions by reducing the dimensionality of the data while retaining key information and allowing for the application of a broader set of ML models. While PCA focuses on capturing the covariance of the data, fPCA accounts for its time-series nature.

The use of PCA or fPCA for dimensionality reduction across various types of high-dimensional datasets has grown substantially [25,26]. Additionally, the combination of PCA or fPCA followed by the application of ML methods has been explored in several fields, including healthcare [27], agriculture [28], and energy [29]. While these studies illustrate the versatility of PCA and fPCA across various disciplines, their application to automated lameness detection in dairy cows is limited.

Finally, another key consideration in how models are developed and validated is to evaluate their generalizability beyond experimental datasets. Unlike other fields, lameness detection requires capturing nuanced behavioral patterns that are farm- and cow-specific. Therefore, models that appear highly accurate in research environments may underperform in clinical settings due to overfitting or inadequate cross-validation practices. Ensuring that models remain reliable across various conditions requires not only better data handling strategies but also more rigorous validation methods to prevent overfitting.

The objectives of this study were 1) to compare the performance of applying ML models to raw accelerometer data and to dimensionally reduced representations via PCA and fPCA, 2) to investigate the impact of different cross-validation strategies on model performance metrics.

## Materials and methods

### 2.1 Overall study design

This study considered 3-dimensional (x, y, and z axes) accelerometer data from dairy cows on Irish pasture-based commercial dairy farms. Three approaches to detecting foot lesions in dairy cows were compared: 1) ML methods applied directly to accelerometer data; 2) ML methods applied to dimensionally reduced, via PCA, accelerometer data; and 3) ML methods applied to dimensionally reduced, via fPCA, accelerometer data. In addition, the effect of different validation approaches was investigated by comparing an *n*-fold cross-validation (nCV) approach and a farm-fold cross validation (fCV) approach.

### 2.2 Selection of cows and farms

The selection of farms, cows, and accelerometers is explained in detail in [30]. Briefly, eleven spring-calving pasture-based dairy herds in Ireland were recruited to participate in a trial during the summer of 2021. Herds were conveniently selected based on proximity to the University College Dublin School of Veterinary Medicine. Prior to visiting each farm, cow identification lists for each herd were extracted from the Irish Cattle Breeding Federation (ICBF) database, and a randomly sampled list of 50 cows to be used in the study was generated with the use of a Microsoft Office Excel random number generator (Microsoft Excel for Microsoft 365, Version 2302). A total of 50 cows were selected per herd, as this was the number of accelerometers available for the study. The data were collected under UCD’s guidelines and approval on ethical animal research (AREC-E-21–28-McAloon). The Research Conduct and Ethics instructions for animal-related research were adhered to during the entire trial period. All farm owners voluntarily participated in the research study.

### 2.3 Accelerometer sensor device

An AX3 Logging 3-axis accelerometer was used to collect the accelerometer data in 3 perpendicular axes, x, y, and z. This was the preferred accelerometer as it gives comprehensive details of the orientation, complex activities, and movements of cows. It also provides efficient battery life for longer-term monitoring and a compact design on-device lithium polymer battery to record up to 21 days of continuous data with 90 minutes of charging time and water resistance up to 1.5 meters; it is thus a suitable accelerometer for a veterinary environment. The collected data was saved in a USB-enabled microcontroller linked to the non-volatile flash memory chip attached at the heart of the AX3 device.

The sampling procedure on each farm consisted of two separate visits, two weeks apart. During the initial visit, the randomly chosen cows were identified, and an accelerometer was fitted to one hind limb; it remained there throughout the trial period. During the second visit to the farm two weeks later, the recruited cows were drafted out from the rest of the herd following milking and foot lesions were recorded for each claw through clinical assessment of each of the four feet and converted to a binary outcome according to severity based on 3 case definitions: case definition 1: any lesion present; case definition 2: moderate lesions present (excluding minor lesions expected to have a low probability of affecting gait); and case definition 3: severe lesions present (only including lesions most likely to result in a detectable gait abnormality). For the purpose of this analysis, we focused on the detection of foot lesions that fulfilled case definition 3 (i.e., severe lesions). No assessment of lameness was performed on the initial visit. Accelerometers were then removed from the animals, and the data downloaded.

### 2.4 Data manipulation and preprocessing

All the pre-processing steps, modelling, and analysis were performed in the R statistical software [31]. Due to the large size of the downloaded data, only the last seven days’ observations were extracted from the original dataset. The last seven days, rather than the first seven days, were considered as foot lesion assessment was conducted on the second visit. To ease computational cost when applying ML methods, the size of the data was further reduced by randomly sampling and retaining one measurement from every 1500 measurements, providing approximately one accelerometer measurement per 30 seconds. The data were then standardised so that each feature had a mean of zero and a standard deviation of one. After these steps, the dataset contained data from *N* = 383 cows, from *f* = 11 farms, each with *p* = 20,000 features per direction. Initially, all three directions (x, y, and z) were considered independently, but our preliminary analysis, based on AUC values, suggested that the z-direction data were optimal for detecting lameness, outperforming the x and y directions’ data. Analysis, therefore, proceeded based on the z direction only.

### 2.5 Approach 1 – ML methods applied to accelerometer data

While many ML methods are not appropriate for use given that the dataset has *N<<p,* random forests (RFs) is one of the few methods that has the potential to handle such datasets as it can be applied directly on the *p = *20,000 features without the need to reduce the number of features *a priori.* Moreover, RFs can capture non-linear patterns and feature interactions, however, this comes at the cost of a higher risk of overfitting and increased computational cost. Here, RFs were applied to the accelerometer data using the “randomForest package” (version 4.7.1.2) [32] in R, with default settings employed.

### 2.6 Approach 2 – ML methods applied to dimension-reduced accelerometer data via PCA

To allow for the application of a wider range of ML models, the dimension of the accelerometer data was reduced. Here, PCA was used to achieve this as it maps high-dimensional data into a lower-dimensional space while preserving as much of the original data’s variance as possible [33,34]. PCA identifies the *q* directions (known as principal components) that capture the most variance in the dataset. The number of components to retain was visually explored using scree plots and determined based on the number required to explain 70% of the variance of the data. Five different ML methods were then applied to the PCA-reduced data: k-nearest neighbours (KNN), random forests (RFs), logistic regression (LR), naïve Bayes (NB), and support vector machines (SVM). Dimension reduction and ML models were applied in R using the “stats” (version 4.3.2) [31]; “class” (version 7.3.22) [35]; “randomForest” [32] and “e1071” (version 1.7.16) [36] packages.

### 2.7 Approach 3 – ML methods applied to dimension-reduced accelerometer data via fPCA

As accelerometer data can be viewed as functional data [37], fPCA was used as an alternative dimension reduction approach. Here, the *pca.fd* function from the “fda” R package (version 6.2.0) [38] was employed to reduce the dimensionality of the data. In this case, the number of components was chosen based on the number required to cumulatively explain 95% of the total variance of the data, again based on the resulting scree plot. The same five ML models and associated settings outlined in Section 2.6 were also applied.

### 2.8 Approaches to cross-validation

Finally, to assess the performance of the three approaches in predicting the presence of foot lesions, we compared two different cross-validation approaches: an *n*-fold CV approach, where *n = 5* and cows were randomly assigned to folds, and a farm-fold CV approach, where cows from the same farm were assigned to the same fold. In the *n*-fold CV approach, the ML models were trained on *n-1 = *4 folds and tested on the held-out fold. This process was repeated *n = 5* times to ensure that each fold was used once as an independent test set. Given the inherent hierarchical structure in the data, i.e., many cows are from the same farm, and to assess the potential utility of predicting lameness in cows in farms not in the training set, it is of interest to see how generalisable results are across farms. Therefore, in the farm-fold approach, given that there was *f* = 11 farms in the data, each of the 11 folds contained data from a single farm. The ML methods were trained on *f*-1 = 10 farm-folds and tested on the remaining farm-fold. This process was repeated *f* = 11 times, ensuring that each farm was used once as an independent test set. For both the *n*-fold and farm-fold CV methods, the mean and standard deviation of the performance metrics (see Section 2.10) across all folds were computed, providing insight into how well the ML methods perform overall.

### 2.9 Performance metrics

We considered severe foot lesions, as diagnosed by the veterinarian, as the target condition or reference test against which we compared the classification algorithms. Several performance metrics were computed to analyze the classification algorithms’ performance, including sensitivity (the proportion of cows with severe foot lesions that were predicted correctly), specificity (the proportion of cows without severe foot lesions that were predicted correctly), negative predictive value (the proportion of cows predicted as negative that did not have severe foot lesions), positive predictive value (the proportion of cows predicted as positive that had foot lesions present), accuracy (the proportion of correctly predicted cows), mean absolute classification error (MACE), balanced accuracy and area under the curve (AUC). We further constructed calibration plots to assess the alignment between the predicted lameness probabilities from our ML models and the proportion of affected cases (observed values). The predicted probabilities were grouped into bins, and for each bin, the mean predicted probability was plotted against the observed proportion of cows with severe foot lesions.

## Results

### Descriptive summary

The initial dataset consisted of approximately 60 million recordings over two weeks in each of the three movement directions (x, y, and z) for each of the 383 dairy cows in 11 dairy herds. After sampling, the dataset was reduced to 20,000 recordings per cow in the z-axis. Dimension reduction using PCA and fPCA reduced the data to *q* = 77 and *q* = 105 components, respectively.

### Comparison of approaches

**Fig 1:**
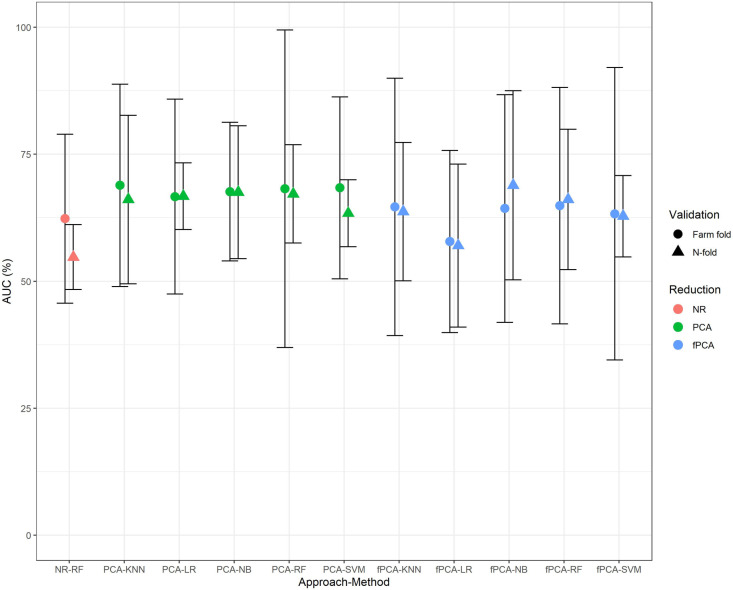
Comparison of mean AUC ( ± 1.96 standard deviations) across all ML approaches with no reduction (NR), PCA and fPCA using n-fold and farm-fold validation methods.

Fig 1 illustrates the influence of dimensionality reduction methods on model performance (as measured by AUC) across *n*-fold CV and farm-fold CV approaches. Overall, PCA-based models appeared to produce better AUC values compared with no reduction and fPCA models. Of the individual methods, PCA combined with KNN produced the highest AUC (mean 69% (sd = 10.71%), based on the farm-fold CV approach), whilst for the validation methods, farm-fold CV produced higher mean AUCs than *n*-fold CV for 8 out of the 11 methods. However, none of these differences were statistically significant, as indicated by the overlapping 95% confidence intervals illustrated in Fig 1 and detailed in the S1 Table. Notably, while the mean AUC is similar across all methods and validation approaches, the variability in performance is higher under a farm-fold approach to validation.

**Fig 2:**
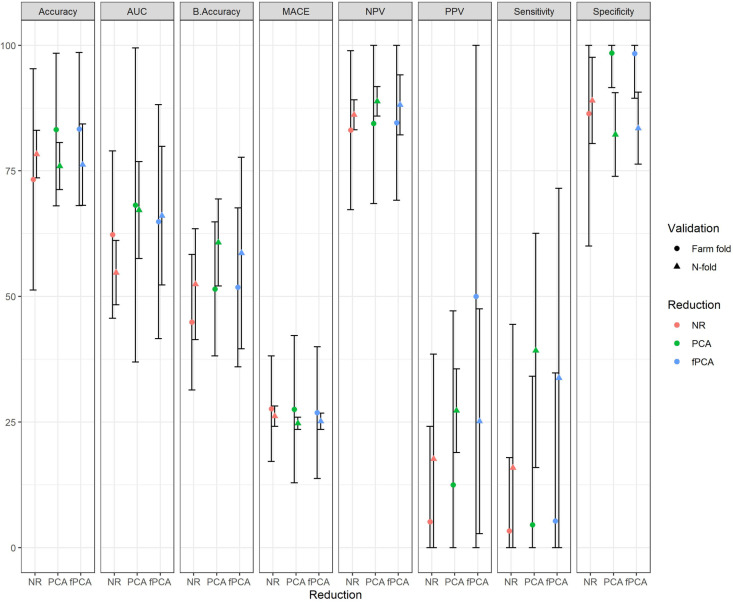
Multiple model performance metrics (mean ± 1.96 standard deviations) for random forest applied to the accelerometer data under the three approaches (NR, PCA, fPCA) and under the two cross validation methods (n-fold and farm-fold).

Secondly, Fig 2 compares the average accuracy, AUC, balanced accuracy, MACE, negative predictive value (NPV), positive predictive value (PPV), sensitivity, and specificity of random forest when applied to non-reduced (NR), PCA-reduced data, and fPCA-reduced data, along with the associated variation. Across all metrics, reduction approaches appeared to have better accuracy, balanced accuracy and sensitivity compared to non-reduced approaches, however these differences were not statistically significant as indicated by the overlapping 95% confidence intervals illustrated in Fig 2 and detailed in the S1 Table. Again, while the mean performance is similar across methods, metrics and validation approaches, the variability in performance is higher under a farm-fold approach to validation.

**Fig 3:**
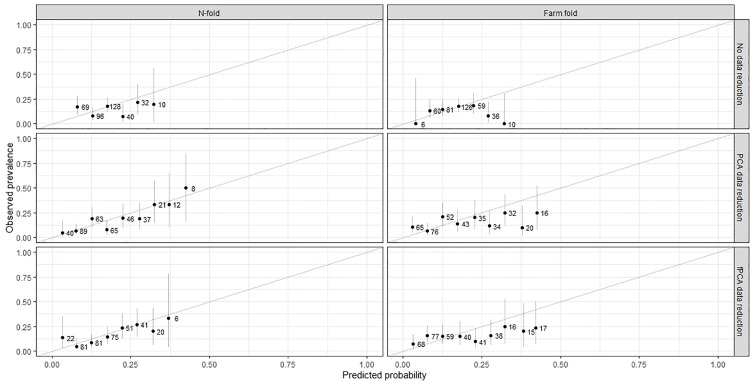
Calibration plots for random forest applied to accelerometer data under three approaches (NR, PCA, fFPCA) and two cross validation methods (n-fold and farm-fold). Dots represent mean per bin with vertical lines indicating 95% confidence intervals. Numbers represent number of animals per bin. Twenty bins were used and those with <5 predicted observations are not plotted.

Furthermore, Fig 3 presents a comparison of calibration plots [39] for the random forest model applied to NR data, PCA-reduced data, and fPCA-reduced data using both *n*-fold and farm-fold cross-validation methods. Notably, the *n*-fold cross-validation approach exhibited better but overoptimistic performance than when predictions were evaluated on a by farm basis. This was particularly evident when RF methods were applied to the reduced data.

## Discussion

We investigated the application of ML methods to accelerometer data for detecting foot lesions in dairy cows. The study focused on the potential utility of dimensionality reduction techniques and the impact of different cross-validation strategies. These aspects were explored to understand how they affect predictive capability and the evaluation of potential generalizability of the models when applied to real-world data.

The set of ML models used in this study was chosen based on their widespread use, accessibility through open-source software, and suitability for handling large-scale data efficiently. The set of ML models considered is known for their strong performance and low computational cost, making them ideal for resource-constrained applications. For more detailed insight into the technical details of the ML methods, the interested reader may refer to [40] and [41].

We found that dimension reduction methods in many cases may improve model performance, whilst having the additional advantage of requiring smaller datasets, bringing reductions in computing time and associated environmental cost, and increasing the number of ML methods that are available to be used. When the number of features is reduced, the performance of the model improves during cross-validation. This improvement is expected, as reducing dimensionality may reduce noise and irrelevant features, thus allowing the model to focus on the most important variables for prediction.

We aimed to detect lameness defined by the presence of foot lesions as opposed to the much more commonly used MS as a gold standard outcome. Across all the methods used in our study, we found that the sensitivity ranged from 3.3% to 39.2%, whilst the specificity ranged from 79.6% to 99.1%. This performance is broadly similar, on average, to reported performance metrics for observer-based gait scoring methods, such as MS which had a sensitivity of 18% to 43% and specificity of 94% to 96% for the detection of foot lesions [30]. However, there is higher variability in the performance metrics under the methods considered here with some low values from poorer-performing methods. In terms of the practical relevance of the methods considered, the best average performance was at the higher end of the performance metrics obtained under observer-led methods, and more robust validation approaches were employed here. In other literature, studies utilizing automated methods for lameness detection have generally been based on the detection of a positive lameness score (i.e., MS above a particular threshold) and have reported varying performance. For example, a camera-based system was developed using cow-specific historical data, achieving a sensitivity of 79% and specificity of 82.3% [42]. In another study, the CattleEye system showed high inter-rater agreement with human assessors, achieving a sensitivity of 52% and specificity of 81% in identifying potentially painful foot lesions [43]. Similarly, a combination of behavioral metrics, milk production, and animal characteristics was used to achieve an AUC of 85%, with both sensitivity and specificity at 78% [44]. Weight shifting between legs was identified as a key predictor of lameness (AUC = 0.71), highlighting its utility for both detection and monitoring of pain relief [45]. More recently, deep learning and image processing techniques were applied, with AdaBoost achieving the highest classification accuracy of 77.9%, demonstrating the potential of machine learning for early lameness detection [11].

These studies underline the capabilities of automated systems in enhancing the accuracy and practicality of lameness detection in dairy herds, illustrating the broader context and potential applications of our findings.

We investigated the impact of different cross-validation methods when assessing model performance by considering *n*-fold and farm-fold CV approaches; the farm-fold approach simulates a real-world farm setting by training models on data from one farm and testing them on data from another. Many studies have tended to focus on an *n*-fold CV approach where animals are randomly sampled from the dataset. However, this approach does not consider clustering of behavior and disease that is likely to occur at the farm level and which might result in over optimistic estimates of model performance and external validity. In contrast, farm-fold CV simulates the likely use of the model in the f + 1 herd and is arguably therefore a more appropriate approach when training and evaluating data from such hierarchical datasets with observations clustered within, in this case, herds. In our study, there were limited differences in performance using different approaches (Figs 1 and 2); however, calibration plots (Fig 3) appeared to demonstrate a more favorable performance for the *n*-fold CV approach. One explanation for this could be that the “by farm” strategy would not allow for farm-specific effects in the test data, making the model less suited to farm-specific variations. In contrast, an *n*-fold CV approach will have included farm variations in the test and training data, potentially inflating measures of predictive performance. One potential way of addressing this issue could be to better account for farm-specific variations (e.g., environmental or management factors), making the approach more robust and applicable to other similar farms. However, this would depend on the extent to which the farm-specific characteristics are shared across farms. Additionally, using methods for prediction that account for the inherent hierarchical structure in the data may result in a more generalizable approach. Recent studies have highlighted the role of diverse cross-validation strategies in improving model evaluation and selection across various fields. For instance, hold-out and n-fold cross-validation methods improved the mean average precision of object detection models deployed on embedded devices like Raspberry Pi, showcasing the impact of sampling techniques on performance [46]. Another study emphasized the importance of cow-independent, experiment-independent, and herd-independent validation schemes for predicting residual feed intake in dairy cows, revealing biases in broader schemes and underscoring the need for realistic assessments and improved calibration [47]. Similarly, researchers explored n-fold, stratified n-fold, and leave-one-out techniques in predicting intrauterine fetal demise, achieving 99% accuracy with gradient boosting and voting classifier models while advocating for black-box evaluation to enhance interpretability [48]. In ecological research, cross-validation approaches were reviewed, with recommendations for leave-one-out or bias-corrected n-fold methods to minimize bias and overfitting, highlighting the importance of predictive scores and careful model specification to avoid parameter estimation bias [49].

### Limitations

While the study highlights several advantages, there are notable limitations. During the initial recruitment of animals, no assessment of foot lesions or lameness scores was performed. As such, the exact onset or duration of lameness over the 2-week data collection period is uncertain. Furthermore, additional data, such as individual animal milk yield, were not available to incorporate into the analysis, potentially limiting the accuracy of predictions [44].

Dimensionality reduction methods, such as PCA and fPCA, rely on assumptions that may not always align with real-world accelerometer data. The choice of the number of dimensions to retain is subjective, which can influence results and model performance. Moreover, even after dimension reduction, there is still a large volume of data to be modelled, not all of which may be relevant for the prediction of lameness. As such, regularized feature selection methods such as LASSO [41] may be useful to simultaneously select relevant features while model fitting, thereby improving prediction performance. Additionally, these methods assume that the sampled data is representative of its time window, but this may not always hold, as the sample might not fully reflect behavior across the entire period. Furthermore, there is uncertainty that the retained dimensions capture information directly related to lameness, as the principal or functional components may retain variation that is not relevant to the trait of interest. Another limitation is that the hierarchical structure of the data, such as measurements from different animals or farms, could violate the assumptions of independence in dimensionality reduction methods.

A key finding of our study was the reduced certainty in estimates when farm-fold cross-validation was used. Farms for this study were recruited based on convenience and may not be representative of herds nationally. Therefore, whilst our key finding regarding the increase in variability of estimates when farm-fold validation was used remains, we cannot necessarily argue that the central estimates for performance metrics (i.e., sensitivity, specificity, AUC, etc.) are accurate for the wider population. Despite these limitations, combining dimensionality reduction with appropriate cross-validation enhances overall performance, reduces computational cost, and expands the suite of methods available to be used. These methods are valuable for high-dimensional datasets, and future research should explore advanced techniques and alternative validation methods to optimize ML models for lameness detection, offering insights into accelerometer-based systems for dairy farming.

## Conclusions

Our study offers practical insights for the dairy industry by highlighting the potential benefits of combining dimensionality reduction with effective cross-validation strategies to improve the performance of ML models applied to wide data, such as accelerometer data. In addition, our study highlights the impact and importance of using data from independent farms. A by-farm approach to cross-validation will likely give a more robust, realistic estimate of general model performance.

While our results do not show sufficient accuracy for field use, they highlight the need for further research in the field of accelerometer-based systems for lameness detection. Future research should aim to carefully utilize the findings of our study to maximize the possibility of developing a suitable and generalizable ML approach for lameness detection.

## Supporting information

S1 Table Confidence intervals for a range of metrics across all reduction, validation and ML methods. (DOCX)
