# Supplementary material for: Comparison of machine learning and validation methods for high-dimensional accelerometer data to detect foot lesions in dairy cattle
Source: PLoS One. 2025 Jun 27;20(6):e0325927. doi: 10.1371/journal.pone.0325927 (PMC12204567; doi:10.1371/journal.pone.0325927)
Supplement: S1 Table — (DOCX) [file pone.0325927.s001.docx]

**Supporting Information**

S1 Table Confidence intervals for a range of metrics across all reduction, validation and ML methods.

| **Reduction method** | **Validation method** | **ML method** | **Metric** | **Estimate (95% confidence interval)** |
| --- | --- | --- | --- | --- |
| PCA | Farm fold | KNN | AUC | 68.88 (48.95,88.81) |
| PCA | n-fold | KNN | AUC | 66.08 (49.48,82.68) |
| fPCA | Farm fold | KNN | AUC | 64.64 (39.3,89.98) |
| fPCA | n-fold | KNN | AUC | 63.7 (50.08,77.32) |
| PCA | Farm fold | LR | AUC | 66.66 (47.49,85.83) |
| PCA | n-fold | LR | AUC | 66.76 (60.19,73.33) |
| fPCA | Farm fold | LR | AUC | 57.82 (39.87,75.77) |
| fPCA | n-fold | LR | AUC | 57.01 (40.96,73.06) |
| PCA | Farm fold | NB | AUC | 67.64 (54,81.28) |
| PCA | n-fold | NB | AUC | 67.51 (54.42,80.6) |
| fPCA | Farm fold | NB | AUC | 64.32 (41.9,86.74) |
| fPCA | n-fold | NB | AUC | 68.91 (50.29,87.53) |
| NR | Farm fold | RF | AUC | 62.32 (45.68,78.96) |
| NR | n-fold | RF | AUC | 54.75 (48.34,61.16) |
| PCA | Farm fold | RF | AUC | 68.21 (36.93,99.49) |
| PCA | n-fold | RF | AUC | 67.2 (57.54,76.86) |
| fPCA | Farm fold | RF | AUC | 64.88 (41.6,88.16) |
| fPCA | n-fold | RF | AUC | 66.09 (52.27,79.91) |
| PCA | Farm fold | SVM | AUC | 68.4 (50.49,86.31) |
| PCA | n-fold | SVM | AUC | 63.38 (56.79,69.97) |
| fPCA | Farm fold | SVM | AUC | 63.28 (34.51,92.05) |
| fPCA | n-fold | SVM | AUC | 62.81 (54.79,70.83) |
| PCA | Farm fold | KNN | Accuracy | 83.83 (66.9,100) |
| PCA | n-fold | KNN | Accuracy | 78.06 (59.95,96.17) |
| fPCA | Farm fold | KNN | Accuracy | 77.55 (60.26,94.84) |
| fPCA | n-fold | KNN | Accuracy | 80.68 (71.27,90.09) |
| PCA | Farm fold | LR | Accuracy | 74.3 (51.02,97.58) |
| PCA | n-fold | LR | Accuracy | 81.21 (77.92,84.5) |
| fPCA | Farm fold | LR | Accuracy | 70.95 (54.94,86.96) |
| fPCA | n-fold | LR | Accuracy | 83.04 (76.18,89.9) |
| PCA | Farm fold | NB | Accuracy | 78.17 (62.69,93.65) |
| PCA | n-fold | NB | Accuracy | 81.7 (73.33,90.07) |
| fPCA | Farm fold | NB | Accuracy | 83.26 (69.68,96.84) |
| fPCA | n-fold | NB | Accuracy | 80.14 (75.28,85) |
| NR | Farm fold | RF | Accuracy | 73.29 (51.26,95.32) |
| NR | n-fold | RF | Accuracy | 78.34 (73.62,83.06) |
| PCA | Farm fold | RF | Accuracy | 83.22 (68.03,98.41) |
| PCA | n-fold | RF | Accuracy | 75.96 (71.28,80.64) |
| fPCA | Farm fold | RF | Accuracy | 83.32 (68.09,98.55) |
| fPCA | n-fold | RF | Accuracy | 76.23 (68.14,84.32) |
| PCA | Farm fold | SVM | Accuracy | 81.5 (70.19,92.81) |
| PCA | n-fold | SVM | Accuracy | 84.47 (81.73,87.21) |
| fPCA | Farm fold | SVM | Accuracy | 82.49 (69.89,95.09) |
| fPCA | n-fold | SVM | Accuracy | 82.51 (78,87.02) |
| PCA | Farm fold | KNN | B.Accuracy | 51.82 (36.47,67.17) |
| PCA | n-fold | KNN | B.Accuracy | 56.24 (39.23,73.25) |
| fPCA | Farm fold | KNN | B.Accuracy | 54.34 (35.48,73.2) |
| fPCA | n-fold | KNN | B.Accuracy | 56.26 (41.64,70.88) |
| PCA | Farm fold | LR | B.Accuracy | 59.27 (32.67,85.87) |
| PCA | n-fold | LR | B.Accuracy | 59.42 (47.39,71.45) |
| fPCA | Farm fold | LR | B.Accuracy | 51.97 (34.53,69.41) |
| fPCA | n-fold | LR | B.Accuracy | 51.57 (45.08,58.06) |
| PCA | Farm fold | NB | B.Accuracy | 49.3 (37.68,60.92) |
| PCA | n-fold | NB | B.Accuracy | 53.67 (37.38,69.96) |
| fPCA | Farm fold | NB | B.Accuracy | 55.51 (39.87,71.15) |
| fPCA | n-fold | NB | B.Accuracy | 55.1 (42.83,67.37) |
| NR | Farm fold | RF | B.Accuracy | 44.88 (31.4,58.36) |
| NR | n-fold | RF | B.Accuracy | 52.45 (41.4,63.5) |
| PCA | Farm fold | RF | B.Accuracy | 51.5 (38.17,64.83) |
| PCA | n-fold | RF | B.Accuracy | 60.74 (52.08,69.4) |
| fPCA | Farm fold | RF | B.Accuracy | 51.83 (36.01,67.65) |
| fPCA | n-fold | RF | B.Accuracy | 58.64 (39.57,77.71) |
| PCA | Farm fold | SVM | B.Accuracy | 55.62 (42.59,68.65) |
| PCA | n-fold | SVM | B.Accuracy | 57.86 (44.18,71.54) |
| fPCA | Farm fold | SVM | B.Accuracy | 53.92 (37.59,70.25) |
| fPCA | n-fold | SVM | B.Accuracy | 52.86 (42.32,63.4) |
| PCA | Farm fold | KNN | MACE | 21.89 (9.13,34.65) |
| PCA | n-fold | KNN | MACE | 22.96 (17.9,28.02) |
| fPCA | Farm fold | KNN | MACE | 21.7 (9.53,33.87) |
| fPCA | n-fold | KNN | MACE | 22.22 (17.22,27.22) |
| PCA | Farm fold | LR | MACE | 27.25 (6.42,48.08) |
| PCA | n-fold | LR | MACE | 23 (21.94,24.06) |
| fPCA | Farm fold | LR | MACE | 29.31 (14.9,43.72) |
| fPCA | n-fold | LR | MACE | 24.5 (23.42,25.58) |
| PCA | Farm fold | NB | MACE | 23.3 (11.07,35.53) |
| PCA | n-fold | NB | MACE | 18.87 (12.75,24.99) |
| fPCA | Farm fold | NB | MACE | 20.44 (8.05,32.83) |
| fPCA | n-fold | NB | MACE | 20.22 (15.2,25.24) |
| NR | Farm fold | RF | MACE | 27.66 (17.15,38.17) |
| NR | n-fold | RF | MACE | 26.18 (24.14,28.22) |
| PCA | Farm fold | RF | MACE | 27.56 (12.9,42.22) |
| PCA | n-fold | RF | MACE | 24.76 (23.54,25.98) |
| fPCA | Farm fold | RF | MACE | 26.9 (13.79,40.01) |
| fPCA | n-fold | RF | MACE | 25.17 (23.54,26.8) |
| PCA | Farm fold | SVM | MACE | 25 (15.24,34.76) |
| PCA | n-fold | SVM | MACE | 23.9 (23.49,24.31) |
| fPCA | Farm fold | SVM | MACE | 25.4 (14.86,35.94) |
| fPCA | n-fold | SVM | MACE | 24.45 (23.12,25.78) |
| PCA | Farm fold | KNN | NPV | 84.44 (68.43,100) |
| PCA | n-fold | KNN | NPV | 87.72 (80.64,94.8) |
| fPCA | Farm fold | KNN | NPV | 85.73 (70.93,100) |
| fPCA | n-fold | KNN | NPV | 87.41 (81.71,93.11) |
| PCA | Farm fold | LR | NPV | 88.82 (75.71,100) |
| PCA | n-fold | LR | NPV | 88.11 (84.33,91.89) |
| fPCA | Farm fold | LR | NPV | 85.38 (70.48,100) |
| fPCA | n-fold | LR | NPV | 85.84 (83.66,88.02) |
| PCA | Farm fold | NB | NPV | 84.35 (69.51,99.19) |
| PCA | n-fold | NB | NPV | 86.43 (82.27,90.59) |
| fPCA | Farm fold | NB | NPV | 85.69 (71.17,100) |
| fPCA | n-fold | NB | NPV | 86.88 (83.06,90.7) |
| NR | Farm fold | RF | NPV | 83.1 (67.26,98.94) |
| NR | n-fold | RF | NPV | 86.16 (83.16,89.16) |
| PCA | Farm fold | RF | NPV | 84.43 (68.48,100) |
| PCA | n-fold | RF | NPV | 88.85 (85.93,91.77) |
| fPCA | Farm fold | RF | NPV | 84.58 (69.15,100) |
| fPCA | n-fold | RF | NPV | 88.12 (82.14,94.1) |
| PCA | Farm fold | SVM | NPV | 86.06 (71.44,100) |
| PCA | n-fold | SVM | NPV | 87.61 (83.89,91.33) |
| fPCA | Farm fold | SVM | NPV | 85.2 (69.19,100) |
| fPCA | n-fold | SVM | NPV | 86.2 (82.71,89.69) |
| PCA | Farm fold | KNN | PPV | 50 (0,100) |
| PCA | n-fold | KNN | PPV | 24.6 (0,64.98) |
| fPCA | Farm fold | KNN | PPV | 24.11 (0,68.37) |
| fPCA | n-fold | KNN | PPV | 26.55 (0,61.46) |
| PCA | Farm fold | LR | PPV | 28.43 (0,79.08) |
| PCA | n-fold | LR | PPV | 31.83 (13.11,50.55) |
| fPCA | Farm fold | LR | PPV | 15.09 (0,46.57) |
| fPCA | n-fold | LR | PPV | 21.97 (7.27,36.67) |
| PCA | Farm fold | NB | PPV | 28.89 (0,100) |
| PCA | n-fold | NB | PPV | 22.89 (0,77.06) |
| fPCA | Farm fold | NB | PPV | 37.3 (0,100) |
| fPCA | n-fold | NB | PPV | 25.27 (6.43,44.11) |
| NR | Farm fold | RF | PPV | 5.16 (0,24.13) |
| NR | n-fold | RF | PPV | 17.67 (0,38.54) |
| PCA | Farm fold | RF | PPV | 12.5 (0,47.15) |
| PCA | n-fold | RF | PPV | 27.28 (18.95,35.61) |
| fPCA | Farm fold | RF | PPV | 50 (0,100) |
| fPCA | n-fold | RF | PPV | 25.14 (2.76,47.52) |
| PCA | Farm fold | SVM | PPV | 48.94 (0,100) |
| PCA | n-fold | SVM | PPV | 43.16 (21.66,64.66) |
| fPCA | Farm fold | SVM | PPV | 21.83 (0,98.37) |
| fPCA | n-fold | SVM | PPV | 20.83 (0,52.11) |
| PCA | Farm fold | KNN | Sensitivity | 4.55 (0,34.11) |
| PCA | n-fold | KNN | Sensitivity | 25.3 (0,88.98) |
| fPCA | Farm fold | KNN | Sensitivity | 21.21 (0,61.63) |
| fPCA | n-fold | KNN | Sensitivity | 21.67 (0,70) |
| PCA | Farm fold | LR | Sensitivity | 37.58 (0,99.99) |
| PCA | n-fold | LR | Sensitivity | 28.64 (1.98,55.3) |
| fPCA | Farm fold | LR | Sensitivity | 24.39 (0,70.18) |
| fPCA | n-fold | LR | Sensitivity | 7.12 (0,30.31) |
| PCA | Farm fold | NB | Sensitivity | 7.12 (0,29.46) |
| PCA | n-fold | NB | Sensitivity | 14.09 (0,45.65) |
| fPCA | Farm fold | NB | Sensitivity | 14.7 (0,50.69) |
| fPCA | n-fold | NB | Sensitivity | 19.7 (0,49.1) |
| NR | Farm fold | RF | Sensitivity | 3.33 (0,17.93) |
| NR | n-fold | RF | Sensitivity | 15.91 (0,44.43) |
| PCA | Farm fold | RF | Sensitivity | 4.55 (0,34.11) |
| PCA | n-fold | RF | Sensitivity | 39.24 (15.94,62.54) |
| fPCA | Farm fold | RF | Sensitivity | 5.3 (0,34.76) |
| fPCA | n-fold | RF | Sensitivity | 33.79 (0,71.52) |
| PCA | Farm fold | SVM | Sensitivity | 17.12 (0,59.77) |
| PCA | n-fold | SVM | Sensitivity | 20.3 (0,53.89) |
| fPCA | Farm fold | SVM | Sensitivity | 11.21 (0,49.57) |
| fPCA | n-fold | SVM | Sensitivity | 10.91 (0,40.19) |
| PCA | Farm fold | KNN | Specificity | 99.09 (93.17,100) |
| PCA | n-fold | KNN | Specificity | 87.18 (55.43,100) |
| fPCA | Farm fold | KNN | Specificity | 87.46 (70.31,100) |
| fPCA | n-fold | KNN | Specificity | 90.85 (71.39,100) |
| PCA | Farm fold | LR | Specificity | 80.96 (49.01,100) |
| PCA | n-fold | LR | Specificity | 90.21 (86.13,94.29) |
| fPCA | Farm fold | LR | Specificity | 79.55 (55.34,100) |
| fPCA | n-fold | LR | Specificity | 96.02 (84.85,100) |
| PCA | Farm fold | NB | Specificity | 91.48 (73.47,100) |
| PCA | n-fold | NB | Specificity | 93.25 (84.53,100) |
| fPCA | Farm fold | NB | Specificity | 96.33 (85.75,100) |
| fPCA | n-fold | NB | Specificity | 90.5 (82.33,98.67) |
| NR | Farm fold | RF | Specificity | 86.43 (60.05,100) |
| NR | n-fold | RF | Specificity | 89 (80.42,97.58) |
| PCA | Farm fold | RF | Specificity | 98.46 (91.6,100) |
| PCA | n-fold | RF | Specificity | 82.24 (73.93,90.55) |
| fPCA | Farm fold | RF | Specificity | 98.35 (89.47,100) |
| fPCA | n-fold | RF | Specificity | 83.49 (76.32,90.66) |
| PCA | Farm fold | SVM | Specificity | 94.12 (76.34,100) |
| PCA | n-fold | SVM | Specificity | 95.42 (88.19,100) |
| fPCA | Farm fold | SVM | Specificity | 96.62 (86.29,100) |
| fPCA | n-fold | SVM | Specificity | 94.8 (85.37,100) |
